# Pilocarpine Injection

**Published:** 1907-06-22

**Authors:** 


### PILOCARPINE INJECTION.

The patient must be in bed. His nightshirt should be removed, and he should then be wrapped closely in a very warm dry blanket, over which two other blankets should be thrown and tucked in well all round the bed. Hot-water bottles, cased in flannel, are put to his feet, and hot drinks may be given by the mouth freely. The pilocarpine is then administered hypodermically, the amount given being usually about $\frac{1}{6}$ grain. The strength of the solution used for injection varies in different hospitals: at St. Thomas's Hospital it is 1 grain of pilocarpine nitrate in distilled water 20 minims, of which the dose is 2 to 6 minims; at Guy's Hospital the strength is 1 grain in 12 minims, with a dosage of 1 to 4 minims. In general practice the pilocarpine nitrate is usually kept ready in hypodermic-tablet form, each tablet containing $\frac{1}{6}$, $\frac{1}{4}$, or $\frac{1}{2}$ grain, and the solution is made with distilled water by the doctor as required. After the injection profuse sweating should begin within half an hour, and may continue for from half an hour to two hours. If free perspiration does not come on, it is advisable to give the patient a tumbler of cold water to drink slowly; the vaso-constriction that results in the splanchnic area serves to drive more blood to the surface of the body and thereby facilitates sweating. The use of pilocarpine is said to be dangerous if diaphoresis does not ensue some authorities hold that everything should be ready for the administration of a hot vapour or hot-air bath, to make certain of the activity of the skin should the pilocarpine fail. In a previous article we have laid stress upon the necessity of knowing that the pilocarpine is really pilocarpine and not pilocarpidine, the test of which is the melting point of the alkaloid. No pilocarpine nitrate should be trusted unless its melting point is known, for so much of that which is supplied contains a large proportion of pilocarpidine.

After the sweating has ceased, but not before, the blankets should be removed gradually, the skin should be rapidly but thoroughly dried, and the patient should be left between fresh, warm, dry blankets.

It is dangerous to give pilocarpine to comatose patients, because not only the sweat and saliva, but also other secretions are much augmented by the drug. This applies to the bronchial secretion, the accumulation of which in the respiratory passages may, in a patient whose reflexes are insensitive, cause serious interference with the action of the heart and lungs.

